# Population-Based 5-Year Follow-Up Study in Taiwan of Dementia and Risk of Stroke

**DOI:** 10.1371/journal.pone.0061771

**Published:** 2013-04-23

**Authors:** Mu-En Liu, Shih-Jen Tsai, Wei-Chiao Chang, Chun-Hung Hsu, Ti Lu, Kuo-Sheng Hung, Wen-Ta Chiu, Wei-Pin Chang

**Affiliations:** 1 Department of Psychiatry, Kaohsiung Veterans General Hospital, Kaohsiung, Taiwan; 2 Department of Psychiatry, Taipei Veterans General Hospital, Taipei, Taiwan; 3 Department of Clinical Pharmacy, School of Pharmacy, Taipei Medical University, Taipei, Taiwan; 4 Department of Healthcare Management, Yuanpei University, Hsinchu, Taiwan; 5 Department of Neurosurgery Center of Excellence for Clinical Trial and Research, Taipei Medical University- Wan Fang Medical Center, Taipei, Taiwan; 6 Graduate Institute of Injury Prevention and Control, Taipei Medical University, Taipei, Taiwan; FuWai hospital, Chinese Academy of Medical Sciences, China

## Abstract

**Background:**

This study estimates the risk of stroke within 5 years of newly diagnosed dementia among elderly persons aged 65 and above. We examined the relationship between antipsychotic usage and development of stroke in patients with dementia.

**Methods:**

We conducted a nationwide 5-year population-based study using data retrieved from the Longitudinal Health Insurance Database 2005 (LHID2005) in Taiwan. The study cohort comprised 2243 patients with dementia aged ≥65 years who had at least one inpatient service claim or at least 2 ambulatory care claims, whereas the comparison cohort consisted of 6714 randomly selected subjects (3 for every dementia patient) and were matched with the study group according to sex, age, and index year. We further classified dementia patients into 2 groups based on their history of antipsychotic usage. A total of 1450 patients were classified into the antipsychotic usage group and the remaining 793 patients were classified into the non-antipsychotic usage group. Cox proportional-hazards regressions were performed to compute the 5-year stroke-free survival rates after adjusting for potentially confounding factors.

**Results:**

The dementia patients have a 2-fold greater risk of developing stroke within 5 years of diagnosis compared to non-dementia age- and sex-matched subjects, after adjusting for other risk factors (95% confidence interval (CI) = 2.58–3.08; *P*<.001). Antipsychotic usage among patients with dementia increases risk of stroke 1.17-fold compared to patients without antipsychotic treatment (95% CI = 1.01–1.40; *P*<.05).

**Conclusions:**

Dementia may be an independent risk factor for stroke, and the use of antipsychotics may further increase the risk of stroke in dementia patients.

## Introduction

The frequency of stroke increases with age. Therefore, these disorders have become major health problems worldwide because the increasing number of elderly and stroke patients contributes to disability in old age [1]. Planning future needs for health services and improved primary and secondary prevention of stroke are critical. Several risk factors such as hypertension, dyslipidemia, obesity, diabetes, and smoking were confirmed to increase the risk of stroke [2]. The relationship between dementia and stroke has attracted significant attention in recent decades. Stroke is known to increase the risk of consequent dementia [3]. However, stroke-free patients with cognitive dysfunction or dementia were suggested to have an increased risk for stroke [4], [5]. A prior population-based prospective study in Italy showed that the relative risks of stroke for elderly persons with moderate and severe cognitive impairment were 1.2 and 2.2 fold compared to those without cognitive impairment, respectively [4]. In Sweden, elderly patients with dementia had a 2.6-fold greater risk of developing stroke after controlling for potential confounders compared to non-dementia participants [5]. Therefore, dementia is currently considered a major risk factor for the development of stroke. However, evidence concerning the risk of stroke in Taiwanese patients with dementia is scarce.

On the other hand, antipsychotics are frequently and increasingly prescribed for the treatment of behavioral and psychological symptoms associated with dementia [6]. This prescription habit changed since the U.S. Food and Drug Administration added warnings of increases in adverse cerebrovascular events to the prescribing information for atypical antipsychotics in 2003 [7]. Previous studies have shown that the use of antipsychotics increased the probability of stroke in elderly patients [8], [9]. Conversely, other studies showed that there was no statistical difference in the risk of cerebrovascular events with conventional and atypical antipsychotic usage compared to the non-user group in dementia patients [10], [11].

Therefore, this nationwide population study was conducted to estimate the relationship between dementia and the subsequent development of stroke within 5 years of follow-up. We further examined whether the use of antipsychotic medications potentially increases the risk of stroke among dementia patients.

## Materials and Methods

### Database

Our study used the data obtained through the Longitudinal Health Insurance Database 2005 (LHID2005), from the National Health Insurance Research Database (NHIRD), which is managed by the Taiwanese National Health Research Institutes (NHRI). These claims data included registration and medical claims for 1 000 000 randomly sampled patients from the total number of NHRI enrollees. The data set contained the entire medical claims data of 1 million beneficiaries from 1996 to 2010. The NHRI informs that there were no statistical differences in age and sex between the sampled group and all enrollees. Because the LHID2005 comprises secondary data (that cannot be used to identify patients) released to the public for research purposes, this study was exempt from full review by the Institutional Review Board. To our knowledge, the LHID2005 data set is one of the largest nationwide population-based databases in the world, and numerous scientific studies are published using its data [12], [13].

### Study Population

The study cohort consists of dementia patients aged 65 years or above who were newly diagnosed with dementia [ICD-9-Code 290.0 (Senile dementia, uncomplicated), 290.1x (Presenile dementia), 290.2x (Senile dementia with delusional or depressive features), 290.3 (Senile dementia with delirium), 290.4x (Arteriosclerotic dementia), 294.1 (Dementia in conditions classified elsewhere), 331.0 (Alzheimer disease), 331.1 (Pick disease), and 331.2 (Senile degeneration of brain)] between January 2003 and December 2005. Cases were only included if patients obtained ≥2 dementia diagnoses in outpatient visits or 1≥ inpatient service because the diagnostic accuracy of the administrative data set was criticized. Between January 2003 and December 2005, the initial diagnosis date of dementia was assigned as the index date for each patient. Patients with dementia before or after the enrollment period and patients who previously experienced strokes (ICD-9-CM codes 430–438) were excluded.

Our comparison cohort was randomly sampled from the remaining subjects of the LHID2005 data set. Subjects were excluded if they were diagnosed with or had a history of any other dementia disease or stroke. The final comparison cohort was chosen from this representative data set through random selection to match a control-to-case ratio of 3 on the basis of age, sex, and index year. Each subject was followed-up for up to five years. The cohort study censored follow-up only on the following conditions: when the subjects expired, on the dates of outcome incidence (i.e. stroke), or at the end of this cohort. Theoretically, loss of follow up would only occur if the patient refused to seek medical service at all upon having a stroke and still stayed in the insurance system for a long time, which is very unlikely.

Several co-variables such as age, sex, hypertension (ICD-9-CM 401.X-405.X), diabetes mellitus (ICD-9-CM 250.X), and hyperlipidemia (ICD-9-CM 272.X) were adopted in our analytical model to examine the relationship between stroke and particular comorbidities. We defined antipsychotics (N05A) according to the Anatomical Therapeutic Chemical (ATC) Classification System [14] and classified dementia patients into 2 groups: treated with antipsychotics or no antipsychotic usage. Exposure to antipsychotics was defined as having been prescribed any antipsychotics for at least 1 day during the follow-up period.

### Level of Urbanization

The Taiwanese NHRI used cluster analysis to examine urbanization levels. They divided 359 towns in Taiwan into 7 strata, with 1 indicating “most urbanized” and 7 indicating “least urbanized”. The categorization of these urbanization levels into 7 clusters were based on Taiwanese census data from 2000. Nevertheless, few dementia cases were in levels 4, 5, 6 and 7; thus, these 4 levels were combined into a single group. Hence, we have 4 strata in urbanization levels.

### Statistical Analysis

All data processing and statistical analyses were performed with the Statistical Package for Social Science (SPSS) software, version 17 (SPSS Inc). Pearson *x* ^2^ tests were used to compare differences in geographic location, monthly income, and urbanization level of patients’ residences between the study group and comparison group. Stratified Cox proportional-hazards regression analysis (stratified by sex, age group, and index year) was performed to examine the risk of subsequent stroke during the 5-year follow-up period for (1) elderly patients with and without dementia, and (2) dementia patients treated with and without antipsychotics. All patients were followed from the index date until presented with stroke or until the end of the 5-year follow-up period. Hazard ratios (HR) and 95% confidence intervals (CI) were adopted to show the risk of stroke. A two-sided *P* value <.05 was considered statistically significant.

## Results

**Figure 1:**
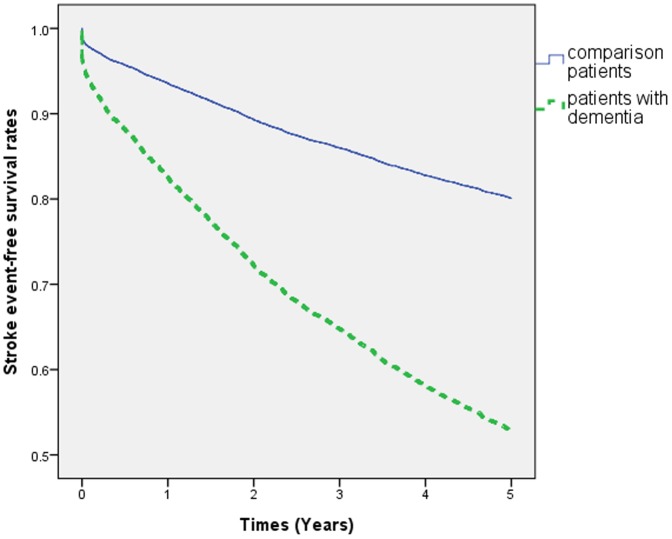
Stroke-free survival rates for patient with dementia and comparison group in Taiwan between 2003 and 2005.

A total of 2243 patients diagnosed with dementia matched the inclusion criteria; 6714 subjects were included in the comparison cohort. The distributions of sociodemographic characteristics and the comorbid medical disorders for the dementia and comparison groups are shown in Table 1. Dementia patients were more vulnerable to hypertension (*P*<.001), diabetes (*P*<.001), and lower monthly income (*P*<.001), and fewer patients resided in the northern part of Taiwan (*P*<.001) compared to controls. During the 5-year follow-up period, 811 dementia patients (36.2% of the study cohort) and 1350 non-dementia subjects (20.1% of the comparison cohort) developed stroke. Cox regression analysis showed that the crude HR of stroke was 2.88 times greater for patients with dementia (95% CI = 2.63–3.14) than for the comparison cohort. The HR remained significant after adjusting for potential confounders (adjusted HR 2.82, 95% CI = 2.58–3.08), as shown in Table 2. The numbers of stroke cases for each year during the 5-year period was shown in Table S1. Cox proportional-hazards regression analysis indicated that dementia patients had significantly lower 5-year stroke-free survival rates (*P*<.001), as shown in Figure 1.

**Table 1:** Demographic Characteristics for the Selected Elders, Stratified by Presence or Absence of Dementia from 2003 to 2005.

	Patients with Dementia(n = 2243)		Patients without Dementia(n = 6714)		P value
	n	%	n	%	
Gender					0.95
Male	1048	46.7	3132	46.6	
Female	1195	53.3	3582	53.4	
AGE(Years), mean (SD)					0.79
> = 65	77.85	(7.16)	77.80	(7.09)	
Follow-up, year, mean (SD)					<0.001
	2.61	(2.34)	4.45	(1.28)	
Urbanization level					0.776
1(most urbanized)	595	26.5	1805	26.9	
2	553	24.7	1582	23.6	
3	338	15.1	1028	15.3	
4(least urbanized)	758	33.7	2299	34.2	
Monthly income					<0.001
No income	791	35.3	2352	35.0	
NT$ 1–15840	630	28.1	1603	23.9	
NT$ 15841–25000	787	35.1	2661	39.6	
NT$≧25001	35	1.6	98	1.5	
Geographic region					<0.05
North	894	39.9	2892	43.1	
Central	623	27.8	1747	26.0	
South	565	25.2	1644	24.5	
Eastern	161	7.2	431	6.4	
Hypertension					<0.001
Yes	1906	85.0	5413	80.6	
No	337	15.0	1301	19.4	
Hyperlipidemia					0.58
Yes	974	43.4	2960	44.1	
No	1269	56.6	3754	55.9	
Diabetes					<0.001
Yes	1147	51.1	2630	39.2	
No	1096	48.9	4084	60.8	
Antipsychotic usage					<0.001
Yes	1450	64.7	1904	28.4	
No	792	35.3	4803	71.6	

**Table 2:** Hazard Ratios (HRs) of Stroke among Dementia Patients during the 5-year Follow-up Period from the Index Ambulatory Visits or Inpatient Care.

	Total		Patients with Dementia		Patients without Dementia	
Development of Stroke	NO.	(%)	NO.	(%)	NO.	(%)
5-year follow-up period						
Yes	2161	24.1	811	36.2	1350	20.1
No	6796	75.9	1432	63.8	5364	79.9
Crude HR (95% CI)				2.88(2.63–3.14)*	1.00	
Adjusted HR (95% CI)				2.82(2.58–3.08)*	1.00	

Total sample number = 8957. Both crude and adjusted HRs were calculated by Cox proportional hazard regressions, and stratified by age and sex. Adjustments are made for age, sex, monthly income, geographic region, hypertension, diabetes, Using antipsychotic drugs.

* Indicates p<0.001.

Dementia patients were divided into 2 groups according to antipsychotic usage for further analysis. A total of 1450 patients had used antipsychotics, and 793 patients had never used antipsychotics. Cox regression model analysis demonstrated that the adjusted HR of stroke was 1.17 times greater for patients with dementia and patients using antipsychotics (95% CI = 1.01–1.40) compared to patients who did not use antipsychotics (Table 3).

**Table 3:** Hazard Ratios (HRs) of Dementia Patients with Antipsychotic/without Antipsychotic during the 5-year Follow-up Period from the Index Ambulatory Visits or Inpatient Care.

	Total		Dementia Patients with using antipsychotic		Dementia Patients without using antipsychotic	
Development of Stroke	NO.	(%)	NO.	(%)	NO.	(%)
Yes	811	36.2	547	37.7	264	33.3
No	1432	63.8	903	62.3	529	66.7
Crude HR (95% CI)			1.18 (1.02–1.35)*		1	
Adjusted HR (95% CI)			1.17 (1.01–1.40)*		1	

Total patients with dementia number = 2243.Adjustments are made for age, sex, monthly income, geographic region, hypertension, diabetes.

* Indicates p<0.05.

## Discussion

The main findings from our study of elderly Taiwanese people may be summarized in the following points: (1) Dementia patients have a more than 2-fold greater risk of developing stroke within 5 years of diagnosis compared to non-dementia age- and sex-matched subjects, after adjusting for other risk factors, and (2) antipsychotic usage increases the risk of stroke in dementia patients.

The association between dementia and stroke originated mainly from community-based studies of people with cognitive dysfunction rather than dementia. A large-scale community- based study in Italy indicated that elevated stroke risk is associated with cognitive impairment [4]. However, cognitive impairment was indicated through a self-administered questionnaire; thus, their results may be different from clinical dementia and confounded by recall bias. Zhu et al [5] showed a 2- to 3-fold greater risk of developing stroke among community-dwelling persons with dementia over a 3-year follow-up period. Their diagnoses of dementia were made according to the Diagnostic and Statistical Manual of Mental Disorders, 3rd edition-revised (DSM III-R). However, the limited number of persons with dementia at baseline (*n = *70) and *short duration* of the *follow*-*up period* may have compromised their findings. This is a nationwide population-based epidemiologic study for examining the relationship between dementia and risk of incident stroke. The strength of this study originates from its nationwide population data set, which included nearly all cases of dementia and stroke in Taiwan during the study period because all practices of psychiatrists and neurologists were covered by the LHID. The large sample size and cohort study design with controls provide considerable statistical power for detecting real and even subtle differences between the 2 cohorts.

Several explanations are plausible for the increased risk of stroke in dementia patients. First, dementia patients have an increased burden of cerebrovascular disease. Several studies have suggested that a significant number of dementia patients suffer from a combination of both degenerative and vascular pathology; thus, a combination of cerebral hypoperfusion, culmination of vascular pathology, and senescence leads to a neuroglial energy crisis, neuronal injury, cognitive impairment, and ultimately, dementia [3]. The cerebrovascular insults may begin numerous years before the manifestation of a clinical stroke. The decline in intellectual level may be correlated with the degree of neurological impairments caused by cerebrovascular insults. Therefore, older people with sufficient deterioration of cognitive function that meet the diagnostic criteria of dementia have a higher probability of suffering an acute stroke.

Second, patients with dementia may be more vulnerable to the complications that are associated with cardiovascular diseases [15]. Furthermore, they may be less likely to recover if affected by these complications compared to non-dementia patients, either because of general frailty, under-diagnosis, under-treatment, and/or noncompliance. Physicians may be hesitant in prescribing several effective drugs such as anticoagulants, even when indicated otherwise because of their potential adverse effects and by regarding that intervention in dementia patients is futile. For example, dementia is generally believed to be a relative contraindication for warfarin treatment in patients with atrial fibrillation [16]. The manner in which physicians behave toward such patients can be affected by the presence of cardiovascular risk factors. Studies have shown that vascular risk factors are treated less often in patients with dementia [17], [18], which may increase the susceptibility to stroke in dementia patients.

On the other hand, we identified that stroke risk with antipsychotic usage was higher among dementia patients compared to patients who did not receive antipsychotics. This finding was consistent with previous reports [19], [20], and provides strong evidence for supporting the US Food and Drug Administration’s warnings of increased stroke risk in elderly dementia patients with exposure to antipsychotics. A crossover study recently using the data of 14 584 patients with incident stroke from the insurance database in Taiwan showed similar findings, indicating that elderly patients treated with antipsychotics had a 1.6-fold greater risk of stroke than patients without prior antipsychotic use [21]. Potential mechanisms for antipsychotic use-related stroke may include orthostatic hypotension in patients with pre-existing cerebrovascular disease, which may lead to “watershed” strokes and antipsychotic-induced tachycardia, causing unstable hemodynamic conditions [22], [23]. Furthermore, over-sedation induced by antipsychotic use may result in dehydration and hemoconcentration, which may be potential mechanisms for increased stroke risk [22]. The association of antipsychotics with stroke may be partially explained by their hyperprolactinemia effect, which may promote platelet aggregation [23], [24]. However, several studies have reported that antipsychotics may inhibit platelet aggregation through serotonin receptor antagonism rather than promote it [25]. Certain observational data have shown that antipsychotics may be associated with an increased risk of venous thromboembolic disease [26]; arterial thrombosis such as stroke shares few risk factors with venous thrombosis. A clear biological mechanism has not been identified for the antipsychotic use-associated increased risk of stroke.

Our study has several limitations. First, all administrative databases are subject to possible coding errors and under- or over-coding problems. The definitions of dementia and stroke relied solely on the coding of hospital diagnoses, but the accuracy of the diagnosis and coding were not verified. Second, information regarding the degree or stage of dementia was unavailable in this database, and the subtypes of dementia were not considered in the analyses of this study. All of these factors may affect the degree of stroke risk. Third, stroke is a heterogeneous disease that includes large-artery atherosclerosis, cardioembolism, small-artery occlusion, and other subtypes [27]. However, patients were only divided into groups of developed stroke or did not develop stroke; thus, we were unable to examine the associations between dementia and stroke subtypes. Fourth, we did not record the duration of treatment (eg, cumulative days of antipsychotic use after initial diagnosis of dementia) for antipsychotics analysis. Moreover, we did not analyze the risk of stroke by the characteristics of antipsychotic use based on average daily dose, classification of antipsychotics, and grouping of the binding affinity for various neurotransmitters; all of these factors may contribute to stroke susceptibility [21]. Finally, individual information regarding dietary habits, cigarette smoking, and body mass index can contribute to stroke, but were not available in the data set. These limitations may have compromised our findings.

We identified dementia as an independent risk factor for stroke using a large-scale population-based study. The use of antipsychotic agents in dementia patients may increase the risk of stroke. Further studies are required to replicate these findings and examine whether preventing cognition decline or discontinuing antipsychotic usage can modify the risk of stroke in dementia patients.

## Supporting Information

Table S1 **The number of stroke cases for each year during the 5-year period.**
(DOCX)
