# Supplementary material for: Population-Based 5-Year Follow-Up Study in Taiwan of Dementia and Risk of Stroke
Source: PLoS One. 2013 Apr 23;8(4):e61771. doi: 10.1371/journal.pone.0061771 (PMC3634021; doi:10.1371/journal.pone.0061771)
Supplement: Table S1 — The number of stroke cases for each year during the 5-year period. (DOCX) [file pone.0061771.s001.docx]

**Table S1**. The number of stroke cases for each year during the 5-year period

| Year | Number of Stroke | Patients with  Dementia | Patients without  Dementia |
| --- | --- | --- | --- |
| 1^st^ Year | 749 | 266 | 483 |
| 2^nd^ Year | 477 | 186 | 291 |
| 3^rd^ Year | 391 | 137 | 254 |
| 4^th^ Year | 284 | 125 | 159 |
| 5^th^ Year | 260 | 97 | 163 |
